# Clinical Implications of Unmasking Dormant Conduction After Circumferential Pulmonary Vein Isolation in Atrial Fibrillation Using Adenosine: A Systematic Review and Meta-Analysis

**DOI:** 10.3389/fphys.2018.01861

**Published:** 2019-01-17

**Authors:** Cheng Chen, Daobo Li, Jeffery Ho, Tong Liu, Xintao Li, Zhao Wang, Yajuan Lin, Fuquan Zou, Gary Tse, Yunlong Xia

**Affiliations:** ^1^Department of Cardiology, First Affiliated Hospital of Dalian Medical University, Dalian, China; ^2^Li Ka Shing Institute of Health Sciences, Faculty of Medicine, Chinese University of Hong Kong, Hong Kong, China; ^3^Tianjin Key Laboratory of Ionic-Molecular Function of Cardiovascular Disease, Department of Cardiology, Tianjin Institute of Cardiology, Second Hospital of Tianjin Medical University, Tianjin, China; ^4^Department of Medicine and Therapeutics, Faculty of Medicine, Chinese University of Hong Kong, Hong Kong, China

**Keywords:** adenosine, dormant conduction, atrial fibrillation, circumferential pulmonary vein isolation, meta-analysis

## Abstract

**Purpose:** Circumferential pulmonary vein isolation (CPVI) is a routine ablation strategy of atrial fibrillation (AF). The adenosine test can be used to unmask dormant conduction (DC) of pulmonary veins after CPVI, thereby demonstrating possible pulmonary vein re-connection and the need for further ablation. However, whether adenosine test could help improve the long term successful rate of CPVI is still controversial. This systemic review and meta-analysis was to determine the clinical utility of the adenosine test.

**Methods:** PubMed, EMBASE, Web of Science and Cochrane Library database were searched through July 2016 to identify relevant studies using the keywords “dormant pulmonary vein conduction,” “adenosine test,” “circumferential pulmonary vein isolation,” and “atrial fibrillation.” A random-effects model was used to compare pooled outcomes and tested for heterogeneity.

**Results:** A total of 17 studies including 5,169 participants were included in the final meta-analysis. Two groups of comparisons were classified: (1) Long-term successful rate in those AF patients underwent CPVI with and without adenosine test [Group A (+) and Group A (−)]; (2) Long-term successful rate in those patients who had adenosine test with and without dormant conduction [Group DC (+) and Group DC (−)]. The overall meta-analysis showed that no significant difference can be observed between Group A (+) and Group A (−) (RR 1.08; 95% CI 0.97–1.19; *P* = 0.16; I^2^ = 66%) and between Group DC (+) and Group DC (−) (RR 1.01; 95% CI 0.91–1.12; *P* = 0.88; I^2^ = 60%).

**Conclusion:** Pooled meta-analysis suggested adenosine test may not improve long-term successful rate in AF patients underwent CPVI. Furthermore, AF recurrence may not be decreased by eliminating DC provoked by adenosine, even though adenosine test was applied after CPVI.

## Introduction

Atrial fibrillation (AF) is a common cardiac arrhythmia, placing a significant burden on healthcare systems worldwide. It has been estimated that 33.5 million people suffering from AF with an increasing prevalence partly attributable to an aging population (Thacker et al., 2013; Chugh et al., 2014). Because pulmonary veins (PVs) are often the triggering sites for initiating and maintaining AF, circumferential PV isolation (CPVI) has been cornerstone of catheter ablation strategy to restore sinus rhythm for AF (Haïssaguerre et al., 2000; Jaïs et al., 2008; Kirchhof et al., 2017). Although feasibility and visibility of the three-dimensional electroanatomic mapping system have been improved, AF recurrence remains a problem due to PV reconnection after CPVI ablation (Ouyang et al., 2005). A study suggested that 20% of AF patients required repeat procedures after a median follow-up of 13 months (Hocini et al., 2005). Previous studies have suggested that PV re-connection can be identified by unmasking dormant conductions (DCs) induced by adenosine (Arentz et al., 2004; Theis et al., 2015; Ghanbari et al., 2016). The adenosine test has been used extensively to identify DCs (Arentz et al., 2004). The mechanism is thought to involve hyperpolarization of the membrane potential of dormant PVs by activating the I_KAdo_ inward rectifier current, which would transiently establish PV reconnection (Datino et al., 2010).

A recent systematic review and meta-analysis has demonstrated a positive outcome on assessment and ablation of dormant conduction (McLellan et al., 2013). However, some of the enrolled studies were based on segmental ablation strategy. Moreover, many studies suggested that whether DCs are associated with high rate of AF recurrence or adenosine test can improve clinical outcome of PVI remains controversial (Elayi et al., 2013; Kobori et al., 2015; Theis et al., 2015; Ghanbari et al., 2016; Kim et al., 2016). Several investigators have attempted to use the appearance of DCs as indication of further ablation using adenosine test after PVI for AF ablation, while results were restricted by low number of participants (McLellan et al., 2013). Therefore, if adenosine test will help to improve ablation success rates after CPVI remains controversial. We conducted this systematic review and meta-analysis to determine the clinical significance of unmasking DCs after CPVI based on long-term follow up using adenosine test as the guidance of extra ablation for AF patients.

## Methods

### Search Strategy

The databases Pubmed, EMBASE, Web of Science and Cochrane library were searched using searching terms and related items including keywords “dormant pulmonary vein conduction,” “adenosine test,” “circumferential pulmonary vein isolation,” and “atrial fibrillation.”

### Inclusion and Exclusion Criteria

The inclusion criteria were limited to articles published in English, involving human subjects of adult age, and published between 2010 and 2016. The exclusion criteria were: (1) ablation for non-AF patients; (2) no adenosine test used; (3) studies including fewer than 90 participants; (4) follow-up period <12 months; (5) CPVI was not used for AF ablation; (6) articles that were case reports, reviews and meta-analyses.

### Study Selection

Data from the different studies were entered in pre-specified spreadsheet in Microsoft Excel. All potentially relevant reports were retrieved as complete manuscripts and assessed for compliance with the inclusion criteria. Two reviewers (C.C. and D.L.) independently reviewed each included study and disagreements were resolved by adjudication with input from a third reviewer (Y.X.). Records matching searching goal were enrolled.

### Data Analysis

The meta-analysis was performed using Review Manager (RevMan 5. 3, Cochrane Collaboration, Oxford, UK). Relative risk (RR) values with 95% confidence intervals (CI) were calculated. Categorical variables were pooled using the Mantel-Hanseal method. The I^2^ statistic from the standard chi-square test (χ^2^), which describes the percentage of the variability in effect estimates resulting from heterogeneity. A fixed effect model was used if I^2^ ≤ 0.25, otherwise the random effect model was used (Higgins and Green, 2011). *P*-value < 0. 05 (two-tailed) was considered statistical significant.

### Quality Assessment

We used the modified Newcastle-Ottawa scale for quality assessment of non-randomized trials and the methodological quality of RCTs was assessed by the components recommended by the Cochrane Collaboration (Higgins and Green, 2011). The quality of each trial except RCTs was quantified by a score of 0–9.

## Results

### Search Results and Study Characteristics

**Figure 1:**
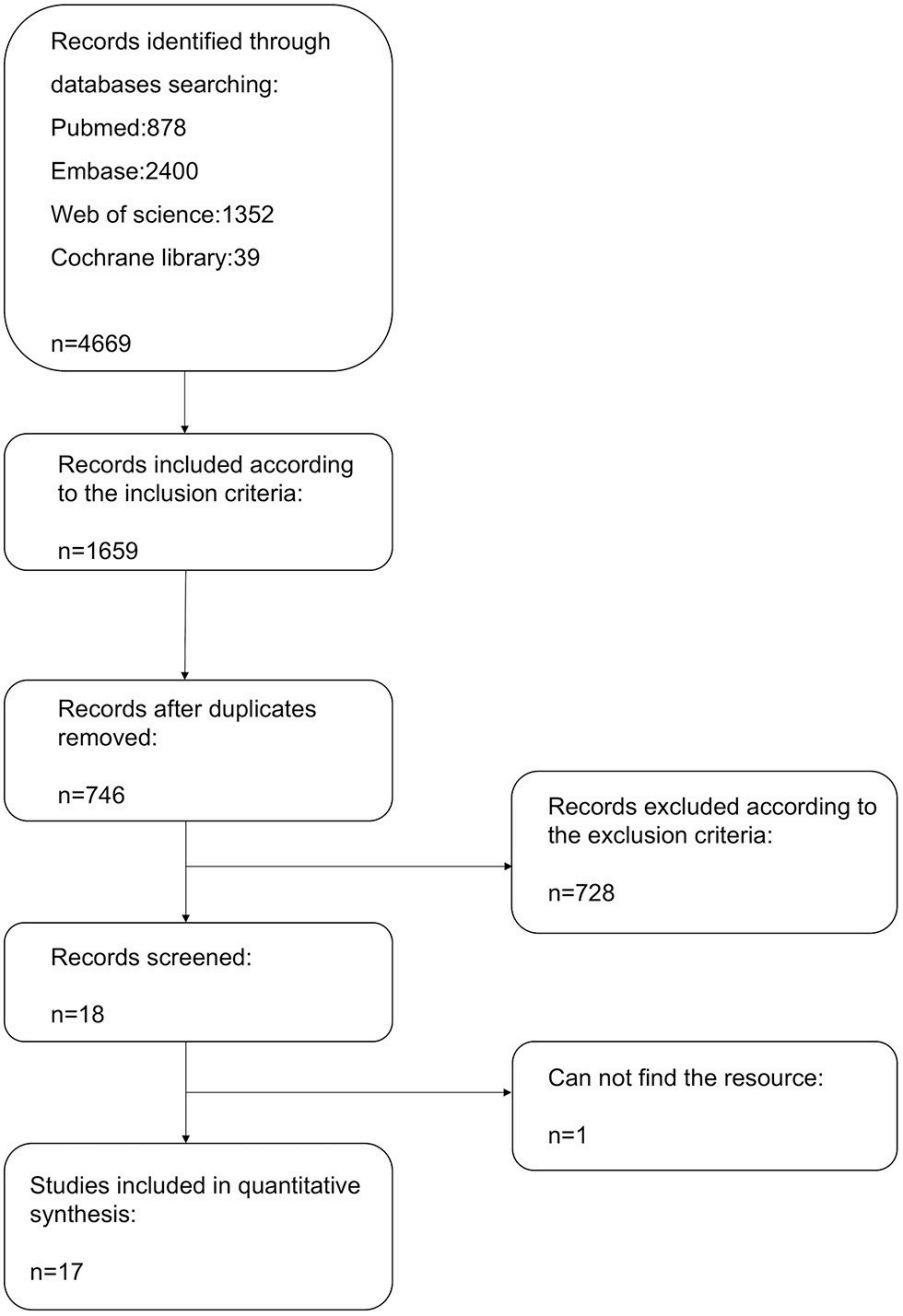
Flow diagram of the study selection process.

A flow diagram detailing the above search terms with inclusion and exclusion criteria is shown in Figure 1. A total of 4,669 records were identified from Pubmed, EMBASE, Web of Science and Cochrane Library databases. Of these, 17 studies met the inclusion criteria and were included in the final meta-analysis (Kumagai et al., 2010; Matsuo et al., 2010; Miyazaki et al., 2012; Van Belle et al., 2012; Cheung et al., 2013; Elayi et al., 2013; Kaitani et al., 2014; Zhang et al., 2014; Compier et al., 2015; Kobori et al., 2015; Kumar et al., 2015; Lin et al., 2015; Macle et al., 2015; Ghanbari et al., 2016; Kim et al., 2016; Tebbenjohanns et al., 2016). Twelve were prospective studies (Matsuo et al., 2010; Van Belle et al., 2012; Elayi et al., 2013; Kaitani et al., 2014; Compier et al., 2015; Kobori et al., 2015; Kumar et al., 2015; Lin et al., 2015; Macle et al., 2015; Theis et al., 2015; Ghanbari et al., 2016; Kim et al., 2016), four were retrospective studies (Kumagai et al., 2010; Matsuo et al., 2010; Miyazaki et al., 2012; Zhang et al., 2014) and four were randomized controlled trials (RCTs) (Kobori et al., 2015; Macle et al., 2015; Theis et al., 2015; Ghanbari et al., 2016). One study used prospective participants as a study group and retrospective cohort as control group (Tebbenjohanns et al., 2016). A total of 5,169 participants were included.

These studies used selective venography or 3-dimensional Electroanatomical Mapping System (including CARTO, Ensite NavX) to identify the PV antrum and subsequently performed CPVI. In four studies, PVI was guided by cryoballoon (second generation cryoballoon, CB-2G) (Van Belle et al., 2012; Compier et al., 2015; Kumar et al., 2015; Tebbenjohanns et al., 2016). The endpoint of electrophysiological study was the presence of entrance block defined by the circular mapping catheter (Lasso, Biosense Webster) or the elimination of all PV potentials or establishment of a bidirectional conduction block between left atrium (LA) and PVs. All participants underwent further ablation if DCs was induced. Two studies described the additional use of superior vena cava isolation (Compier et al., 2015; Kumar et al., 2015).

In this meta-analysis, we supposed to determine: (1) if adenosine test would help to increase the success rate of PVI; and (2) furthermore, if DCs induced by adenosine play an important role in AF recurrence after CPVI. Hence, in the first part, Group A (+) and Group A (−) were divided according to whether adenosine was administrated or not. And in the second part, Group DC (+) and Group DC (−) were divided according to whether the DCs appeared or not after adenosine administration. All of DCs induced by adenosine test in Group A (+) and Group DC (+) patients were eliminated after CPVI. The baseline characteristics of these studies are listed in Table 1, and those of procedure parameter are shown in Table 2. Quality assessment of the included studies was made using the Newcastle–Ottawa Scale for non-randomized case–control studies and the Cochrane Collaboration's tool for randomized trials (Table 3).

Table 1ABasic information and operation details in Group A (+) and Group A (−).

**Table:** 

**Article**	**Comparator** **groups**	**Publish** **year**	**Center**	**Study** **type**	**Electroanatomic** **mapping system**	**Type of AF** ablation^*^	**Ablation** **endpoint**	**RF** energy^*^	**MPT** (min)^*^	**MFT** (min)^*^
Kobori et al., 2015	ATP guided PVI Conventional PVI	2015	Multiple	Prospective RCT^*^	CARTO, Ensite NavX	CPVI^*^	Disappearance of DC in ATP-guided PVI group^*^	35 W^*^ (limited to 20 W on the posterior wall)	195 192	58.4 58.0
Theis et al., 2015	Adenosine group Control group	2015	Single	Prospective RCT	Ensite NavX	Standardized PVI procedure^*^	Elimination of PV potentials recorded on circumferential PV catheter^*^	Maximum power 30 W	126 ± 45	23 ± 9
Elayi et al., 2013	Group 1 Group 1A Group 1B Group 1C Control Group 2	2013	Single	Prospective CT^*^	Lasso, Lasso 2515, Biosense-Webster	PVAI, SVC was also isolated by ablation of the sharp SVC potentials^*^	Electrical isolation of the PV antrum region^*^	30–35 W on the posterior wall; 40–45 W at other locations	–	60 ± 24 53 ± 17
Ghanbari et al., 2016	Adenosine No adenosine	2016	Single	Prospective RCT	CARTO, Biosense-Webster	Encircle PV ostia	–	25 W	216.8 ± 60.6 202.0 ± 62.5	33.6 ± 13.4 32.1 ± 13.2
Kumagai et al., 2010	ATP group Control group	2010	Single	Retrospective analysis	BeeAT, Japan Lifeline Co., Ltd., Japan	Circumferential ablation	Creation of bidirectional conduction block	≤35 W and ≤30 W on sites near the esophagus	–	–
Compier et al., 2015	Adenosine + group Adenosine – group	2015	Single	Prospective CT	Lasso 2515 catheter	PVI guided by cryoballoon and circular mapping catheter	Entrance and exit was block	–	57 + 21 51 + 11	24 + 11 34 + 10
Kumar et al., 2015	Adenosine group Non-adenosine group	2015	Single	Prospective CT	–	Inner lumen endoluminal spiral catheter, CB-2G balloon guided PVI^*^	Twice 4 min applications of each PV and there was entrance and exit block after adenosine test	–	174 + 44 212 + 47	34 + 13 40 + 18
Van Belle et al., 2012	Adenosine group No adenosine group	2012	Single	Prospective CT	A circular mapping catheter	28 mm, 12 Fr cryoballoon catheter	–	–	202 ± 68	41 ± 24
Tebbenjohanns et al., 2016	Study group Control group	2016	Single	Prospective retrospective	A spiral mapping catheter	CB^*^ catheter	–	–	78 + 12 93 + 12	14 + 3 14 + 4

**Table:** 

**Article**	**Comparator groups**	**ATP (dose/period time)**	**Follow up(m)**	**Follow up(method)**	**Free form AF** ***n*** **(%)**	**P**	Conclusion^*^
Kobori et al., 2015	ATP guided PVI Conventional PVI	0.4 mg/kg without waiting period	15	12-lead electrocardiogram, one-channel electrocardiogram, ambulatory electrogram recorder, 24 h Holter monitoring	79.2% 76.9%	Primary endpoint 0.09 secondary efficacy endpoint 0.07	–
Theis et al., 2015	Adenosine group Control group	≥10-mg adenosine, incremental values increased by 5-mg steps	24.8 ± 4.01 29.16 ± 4.87	48-h Holter-ECGs, ECG^*^	88% 92%	0.001 (overall follow-up)	+
Elayi et al., 2013	Group 1 Group 1A Group 1B Group 1C Control Group 2	Intravenous injection of 12 mg. ISP infusion was started: 5 mcg for 3 min, then 10 mcg for 3 min, 15 mcg for 3 min, 20 mcg for 3 min, and 30 mcg for 3 min	22 ± 8	48-h Holter monitors, event recorder	–	Groups 1A/1B and 1B/1C (*P* < 0.001) groups 1A and group 1C (*P* = 1.0) groups 1 and groups 2 (*P* = 0.038)	+
Ghanbari et al., 2016	Adenosine No adenosine	6–24 mg adenosine ISP infused at rates of 5, 10, 15, and 20 μg/min for 2 min at each infusion rate in each group as above	9.2 ± 7	Auto-triggered event recorder	24/61 [39.3%] 23/68 [33.8%]	0.83	–
Kumagai et al., 2010	ATP group Control group	10 mg ATP administered during an intravenous ISP infusion (5 μg/ min)^*^	16 ± 5.2 16 ± 6.9	ECG, 24-h Holter monitoring	76.4% 62.3%	0.03	+
Compier et al., 2015	Adenosine + group Adenosine – group	Adenosine initiated at 6/12 mg, increased up to 30 mg until at least one atrial beat with AV-block was observed with 30-min waiting period	12 + 1 11 + 1	ECG, 24 h Holter	64% 47%	0.02	+
Kumar et al., 2015	Adenosine group Non-adenosine group	Waiting time of 30 min, 12–15 mg adenosine	13 + 1 12 + 2	–	84% 79%	0.06	–
Van Belle et al., 2012	Adenosine group No adenosine group	25 mg adenosine	17 ± 5	ECG, 24-h Holter recording, a symptom questionnaire, Transtelephonic Holter monitoring	23 (68%) 29 (46%)	0.04	+
Tebbenjohanns et al., 2016	Study group Control group	A bolus of adenosine with a short duration	15 ± 3.6	24-h Holter monitoring and external event recording	81% 79%	NS	–

* Parts of values represent mean ± difference. Conclusion^*^:(+) represents experimental group and controlled group have significant difference;(−) represents experiment group and controlled group have no significant difference. PVI^*^, pulmonary vein isolation; CPVI^*^, circumferential pulmonary vein isolation; PV^*^, pulmonary vein; RCT^*^, randomized controlled trial; DC^*^, dormant conduction; CT^*^, clinical trial; ISP^*^, isoproterenol; MPT^*^, mean procedure time; MFT^*^, mean fluoroscopic time; AF^*^, atrial fibrillation; RF^*^, radiofrequency; SVC^*^, superior vena cava; ECG^*^, electrocardiograph; CB-2G^*^, second-generation cryoballoon; CB, cryoballoon; W^*^, watt.

Table 1BBaseline information in Group A (+) and Group A (−).

**Table:** 

**Article**	**Comparator groups**	**Numbers of Sample**	**Numbers of group**	**Age**	**Male *n* (%)**	PSAF^*^n (%)	**AF history**	LAD^*^ (mm)	HP^*^ (*n*/%)
Kobori et al., 2015	ATP guided PVI Conventional PVI	2120	1112 1001	58.6+8.6 68.5+8.8	856 (77.0) 723 (72.7)	737 (66.3) 683 (68.2)	23.3 [8.8–60.8] m 26.4 [9.4–67.5] m	38.9 + 6.3 39.2 + 6.2	535 (47.6%) 590 (58.9%)
Theis et al., 2015	Adenosine group Control group	152	76 76	63 ± 10 64 ± 9.11	45 (59) 33 (43)	152 (100%)	–	22.17 ± 5.18 cm^2^ 23.24 ± 4.81 cm^2^	46 (61) 53 (70)
Elayi et al., 2013	Group 1 Group 1A Group 1B Group 1C Control Group 2	388	32 83 74 196	63.5 ± 10.5 63.6 ± 10.1 63.9 ± 10.4 63.6 ± 10.2	20 (62%) 54 (65%) 58 (78%) 150 (76%)	3 (10%) 11 (13%) 12 (16%) 30 (15%)	4.7 ± 3.7 y 4.6 ± 4 y 4.4 ± 3.8 y 4.7 ± 4.1 y	46.3 ± 4.3 46.0 ± 4.2 45.8 ± 4.2 46.3 ± 4.3	15 (47%) 39 (47%) 32 (43%) 93 (48%)
Ghanbari et al., 2016	Adenosine No adenosine	129	61 68	59.7 ± 8.7 58.9 ± 10.7	37 (60.6%) 53 (77.9%)	129 (100%)	–	41.0 ± 5.3 41.2 ± 6.4	33 (54.1%) 28 (45.9%)
Kumagai et al., 2010	ATP group Control group	212	106 106	58 ± 11 59 ± 10	70.0% 78.3%	94 86	4.5 ± 3.9 y 5.0 ± 5.5 y	39.4 ± 5.4 39.7 ± 5.7	21.7 20.0
Compier et al., 2015	Adenosine + group Adenosine – group	98	36 62	61 + 10 59 + 11	78% 70%	86% 90%	64 + 60 m 58 + 53 m	42 + 6.7 42 + 5.6	50 52
Kumar et al., 2015	Adenosine group Non-adenosine group	90	45 45	57.4 + 9.5 56.6 + 11.2	27 34	40 39	8 + 7.1 y 7 + 3.8 y	LA volume: 72 + 14 cc 77 + 18 cc	14 (31%) 18 (40%)
Van Belle et al., 2012	Adenosine group No adenosine group	99	34 65	57 ± 12 57 ± 12	24 46	34 65	7 ± 5 y 7 ± 6 y	45 ± 7 42 ± 6	–
Tebbenjohanns et al., 2016	Study group Control group	192	53 139	66 + 10 61 + 11	27 75	38 (72%) 87 (63%)	6 + 4 y 5 + 3 y	40 + 6 41 + 7	–

**Table:** 

**Article**	**Comparator groups**	**Ischemic heart disease**	**Diabetes (n/%)**	LVEF^*^ **(%)**
Kobori et al., 2015	ATP guided PVI Conventional PVI	17 (1.5%) 20 (2.0%)	141 (12.7%) 141 (14.1%)	64.2 + 7.9 64.6 + 7.3
Theis et al., 2015	Adenosine group Control group	–	–	54.74 ± 1.61 55
Elayi et al., 2013	Group 1 Group 1A Group 1B Group 1C Control Group 2	5 (15%) 13 (16%) 15 (20%) 40 (20%)	4 (12%) 8 (10%) 11 (15%) 25 (13%)	54 ± 12 55 ± 9 57 ± 11 55 ± 9
Ghanbari et al., 2016	Adenosine No adenosine	–	6 (9.8%) 8 (11.8%)	59.7 ± 5.4 59.3 ± 5.6
Kumagai et al., 2010	ATP group Control group	–	–	65.1 ± 8.9 63.8 ± 9.6
Compier et al., 2015	Adenosine + group Adenosine – group	–	–	–
Kumar et al., 2015	Adenosine group Non-adenosine group	9 (20%) 8 (18%)	–	56 + 6 57 + 8
Van Belle et al., 2012	Adenosine group No adenosine group	–	–	–
Tebbenjohanns et al., 2016	Study group Control group	–	–	–

* Abbreviations as per Table 1A. PSAF^*^, paroxysmal atrial fibrillation; LAD, left atrial diameter; HP, hypertension; LVEF, left ventricular ejection fraction.

Table 2ABasic information and operation details in Group DC (+) and Group DC (−).

**Table:** 

**Article**	**Comparator groups**	**Publish year**	**Center**	**Study type**	**Electroanatomic mapping system**	**Type of AF ablation**	**Ablation end point**	**RF energy**	**MPT (min)**	**MFT (min)**
Zhang et al., 2014	ATP (+) Group ATP (−) Group	2014	Single	Retrospective analysis	CARTO	Standard CPVI procedure by irrigated tip catheter	Entrance block	–	–	–
Kim et al., 2016	Dormant conduction No dormant conduction	2016	Single	Prospective CT	CARTO	4-mm open irrigated catheter, CPVI	No PV potentials recorded by the circular mapping catheter. Exit block was confirmed when PV to LA dissociation was observed during PV pacing^*^	25–35 W	194.0 ± 55.4	67.9 ± 51.9
Kaitani et al., 2014	DC – group DC+ group	2014	Multiple	Prospective observational study	CARTO XP, NavX	CPVI by irrigated-tip catheters	Entrance block as shown by t elimination of the superior and inferior pulmonary vein potentials	20–40 W	–	–
Macle et al., 2015	Adenosine + No further ablation Adenosine + Ablation until adenosine – Adenosine – Registry group Adenosine – routine follow-up	2015	Multiple	Prospective RCT	–	Circumferential ablation at the PV ostia by the circular catheter	PV spikes are no longer recorded	–	–	–
Matsuo et al., 2010	Group A: dormant PV conduction [+] Group B: dormant PV conduction [–]	2010	Single	Retrospective analysis	CARTO	Circular catheter venography was performed	Establishment of a bidirectional conduction block between LA and PV	25–35 W	220 ± 71 217 ± 65	125 ± 43 132 ± 54
Miyazaki et al., 2012	Group-1:no adenosine response Group-2: adenosine response	2012	Single	Retrospective analysis	CARTO	Circumferentially extensively ablated by circular mapping catheters	The elimination of all PV potentials	35 W	–	–
Cheung et al., 2013	Dormant conduction [+] group Dormant conduction [-] group	2013	Single	Prospective CT	CARTO or EnsiteNavX	Circumferential ablation	(1) Entrance block or abolition of PV Potentials (2) Exit block with absence of left atrial capture of the circular mapping catheter	45 W (<30 W on the posterior wall)	–	–
Lin et al., 2015	Dormant conduction group No Dormant conduction group	2015	Single	Prospective CT	CARTO or EnsiteNavX	Circumferential ablation	(1) Entrance block or abolition of PV potentials (2) Exit block with absence of left atrial capture of the circular mapping catheter	15–50 W	–	–

**Table:** 

**Article**	**Comparator groups**	**ATP (dose/period time)**	**Follow up (m)**	**Follow up (method)**	**Free form AF** ***n*** **(%)**	***P***	**Conclusion**
Zhang et al., 2014	ATP (+) Group ATP (−) Group	ATP 40 mg during an intravenous ISP infusion (5 μg/min)	18.7 ± 6.4	–	30/39 (76.9%) 176/261 (67.3%)	ATP (+-) vs. ATP (++) *p* = 0.02	+
Kim et al., 2016	Dormant conduction No dormant conduction	20 mg If dormant conduction was observed, 12 and 6 mg adenosine were injected serially and dormant conduction was observed to identify the adequate adenosine dose	12	24-h Holter monitoring	74.8% 72.6%	0.9	–
Kaitani et al., 2014	DC – group DC+ group	A continuous isoproterenol infusion (0.5–2 mg/min) at begin. A waiting period of at least 15 min,40 mg ATP	27.1 ± 15	ECG, Holter, an event recorder	66.7% 80.0%	0.12	–
Macle et al., 2015	Adenosine + No further ablation Adenosine + Ablation until adenosine – Adenosine – Registry group Adenosine – routine follow-up	12 mg ATP 20 min observation period	12.3	Holter	51 (37.2%) 88 (59.9%) 56 (48.7%) —	①vs. 0.0002② ①vs. 0.0421③ ②vs. 0.0639③	+
Matsuo et al., 2010	Group A: dormant PV conduction [+] Group B: dormant PV conduction [–]	20 mg of ATP under ISPl infusions	30 ± 13	Electrocardiogram recordings 24-h ambulatory monitoring	125 (89.9%) 86 (91.5%)	0.79	–
Miyazaki et al., 2012	Group-1: no adenosine response Group-2: adenosine response	40 mg during intravenous ISP infusion	12	ECG, Holter, event recorder	72.8% 51.3%	0.03	+
Cheung et al., 2013	Dormant conduction [+] group Dormant conduction [–] group	12-mg adenosine was injected followed by 20 mL saline.	12.5	7–14 days continuous mobile telemetry monitors	64% 76%	0.062	–
Lin et al., 2015	Dormant conduction group No Dormant conduction group	A-12 mg adenosine was injected followed by 20 cc of saline with escalating doses of 18 mg and 24 mg if atrioventricular block was not observed.	20 ± 9	7–14 days continuous mobile telemetry monitors; telephone follow-up for symptoms	47% 61%	0.12	–

* Abbreviations as per Table 1A. LA^*^, left atrial.

**Table 2B:** Baseline information in Group DC (+) and Group DC (−).

**Article**	**Comparator groups**	**Numbers of sample**	**Numbers of group**	**Age**	**Male *n* (%)**	**PSAF *n* (%)**	**AF history**	**LAD (mm)**	**HP (%)**	**Ischemic heart disease**	**Diabetes**	**LVEF (%)**
Zhang et al., 2014	ATP (+) Group ATP (−) Group	300	39 261	52.7 ± 4.9 54.4 ± 6.7	19 125	300 (100%) –	3.2 ± 0.6 y 3.7 ± 0.4 y	37.4 ± 3.4 36.8 ± 4.2	–	–	–	61.4 ± 2.7 62.2 ± 3.6
Kim et al., 2016	Dormant conduction No dormant conduction	378	92 286	60.7 ± 11.3 60.2 ± 11.1	69 186	49 (53.3%) 151 (52.8%)	–	43.7 ± 12.6 43.1 ± 13.5	44 (47.8%) 146 (51.0%)	–	17 (18.5%) 43 (15.0%)	–
Kaitani et al., 2014	DC – group DC+ group	110	75 35	62.5 + 9.8 61.8 + 9.2	55 26	–	45.9 ± 40 m 59.3 ± 7 m	38.2 + 6 38.7 + 0.5	49 (65.3%) 22 (62.9%)	–	8 (10.7%) 4 (11.4%)	–
Macle et al., 2015	Adenosine + No further ablation Adenosine + Ablation until adenosine – Adenosine – Registry group Adenosine – routine follow-up	550	137 147 117 133	58.4 60.2 58.9 60.4	97 108 87 86	100%	3.4 y 4.0 y 3.0 y 4.0 y	39.6 40.1 40.1 39.9	50 (36.5%) 62 (42.2%) 54 (46.2%) 54 (40.6%)	15 (10.9%) 16 (10.9%) 10 (8.5%) 14 (10.5%)	6 (4.4%) 8 (5.4%) 11(9.4%) 8 (6.0%)	60.1 59.9 59.1 60.2
Matsuo et al., 2010	Group A: dormant PV conduction [+] Group B: dormant PV conduction [–]	233	139 94	54.3 ± 9.6 54.2 ± 10.9	122 84	89 55	4.5 ± 4.0 y 4.3 ± 3.7 y	38.5 ± 5.5 39.7 ± 5.7	31 (22.3%) 27 (28.7%)	–	–	65.9 ± 6.6 65.8 ± 7.4
Miyazaki et al., 2012	Group-1: no adenosine response Group-2: adenosine response	109	70 39	61.4 ± 11.2 59.4 ± 10.3	58 33	109 (100%)	60.7 ± 59.1 m 57.4 ± 43.9 m	38.1 ± 5.4 39.4 ± 5.5	24 (34%) 16 (41%)	–	–	65.8 ± 8.3 66.4 ± 9.0
Cheung et al., 2013	Dormant conduction [+] group Dormant conduction [–] group	152	44 108	62 ± 9 60 ± 11	34 86	29 (66%) 67 (62%)	29 (66) 67 (62)	4.0 ± 0.6 4.3 ± 0.7	23 (52%) 42 (39%)	–	7 (16%) 11 (10%)	60 ± 11 59 ± 11
Lin et al., 2015	Dormant conduction group No Dormant conduction group	152	45 107	61 ± 9 59 ± 11	35 85	30 (67%) 66 (62%)	30 (67) 66 (62)	–	23 (51%) 41 (38%)	–	7 (16%) 11 (10%)	60 ± 10 59 ± 11

* *Abbreviations as per Table 1A*.

**Table 3A:** Assessment of the quality of included studies in Group A (+) and Group A (−)^*^.

**Study**	**Assessment**	**Classification (attributable stars)**
Kobori et al., 2015	Unclear risk of selection bias (insufficient information about the sequence generation and allocation concealment); Unclear risk of performance bias (insufficient information about blinding of participants and personnel); Unclear risk of detection bias (insufficient information about blinding of outcome assessment); low risk of attrition bias (complete outcome for all the patients enrolled); Unclear risk of reporting bias (insufficient information about selective reporting); Unclear risk of other bias (insufficient information about other sources of bias).	–
Theis et al., 2015	Unclear risk of selection bias (insufficient information about the sequence generation and allocation concealment); Unclear risk of performance bias (insufficient information about blinding of participants and personnel); Unclear risk of detection bias (insufficient information about blinding of outcome assessment); low risk of attrition bias (complete outcome for all the patients enrolled); Unclear risk of reporting bias (insufficient information about selective reporting); Unclear risk of other bias (insufficient information about other sources of bias).	–
Elayi et al., 2013	Adequate case definition; consecutive series of cases; hospital controls; adequate information concerning the selection and definition of controls; groups controlled for all the baseline characteristics; ascertainment of outpatient exposure to adenosine text based on medical records for experiment groups; patients not blinded to case–control status.; same non-Response rate for both groups.	6
Ghanbari et al., 2016	Low risk of selection bias (treatment assignment was concealed in numbered, sealed envelopes, the research staff opened the envelope and revealed the randomization assignment in the electrophysiology laboratory and insufficient information about the sequence generation); Unclear risk of performance bias (insufficient information about blinding of participants and personnel); Unclear risk of detection bias (insufficient information about blinding of outcome assessment); low risk of attrition bias (complete outcome for all the patients enrolled); Unclear risk of reporting bias (insufficient information about selective reporting); Unclear risk of other bias (insufficient information about other sources of bias).	–
Kumagai et al., 2010	Adequate case definition; consecutive series of cases; hospital controls; adequate information concerning the selection and definition of controls; groups controlled for all the baseline characteristics; ascertainment of outpatient exposure to adenosine text based on medical records for experiment groups; patients not blinded to case–control status.; same non-Response rate for both groups.	6
Compier et al., 2015	Adequate case definition; consecutive series of cases; hospital controls; adequate information concerning the selection and definition of controls; groups controlled for all the baseline characteristics; ascertainment of outpatient exposure to adenosine text based on medical records for experiment groups; patients not blinded to case–control status.; same non-Response rate for both groups.	6
Kumar et al., 2015	Adequate case definition; consecutive series of cases; hospital controls; adequate information concerning the selection and definition of controls; groups controlled for all the baseline characteristics; ascertainment of outpatient exposure to adenosine text based on medical records for experiment groups; patients not blinded to case–control status.; same non-Response rate for both groups.	6
Van Belle et al., 2012	Adequate case definition; consecutive series of cases; hospital controls; adequate information concerning the selection and definition of controls; groups controlled for all the baseline characteristics except the LA^*^ diameter; ascertainment of outpatient exposure to adenosine text based on medical records for experiment groups; patients not blinded to case–control status.; same non-Response rate for both groups.	5
Tebbenjohanns et al., 2016	Adequate case definition; consecutive series of cases; hospital controls; adequate information concerning the selection and definition of controls; groups controlled for all the baseline characteristics except the age and history with AF^*^,; ascertainment of outpatient exposure to adenosine text based on medical records for experiment groups; patients not blinded to case–control status.; same non-Response rate for both groups.	5

* Assessment of the quality of included studies according to Newcastle–Ottawa Scale for nonrandomized case–controls studies and the Cochrane Collaboration's tool for assessing risk of bias in randomized trials; ^*^LA, left atrial; ^*^AF, atrial fibrillation.

**Table 3B:** Assessment of the quality of included studies in Group DC (+) and Group DC (−)^*^.

**Study**	**Assessment**	**Classification (attributable stars)**
Zhang et al., 2014	Adequate case definition; consecutive series of cases; hospital controls; adequate information concerning the selection and definition of controls; groups controlled for all the baseline characteristics; ascertainment of outpatient exposure to adenosine text based on medical records for experiment groups and controls; patients not blinded to case–control status.; same non-Response rate for both groups.	7
Kim et al., 2016	Adequate case definition; consecutive series of cases; hospital controls; adequate information concerning the selection and definition of controls; groups controlled for all the baseline characteristics; ascertainment of outpatient exposure to adenosine text based on medical records for experiment groups and controls; patients not blinded to case–control status.; same non-Response rate for both groups.	7
Kaitani et al., 2014	Adequate case definition; consecutive series of cases; hospital controls; adequate information concerning the selection and definition of controls; groups controlled for all the baseline characteristics; ascertainment of outpatient exposure to adenosine text based on medical records for experiment groups and controls; patients not blinded to case–control status.; same non-Response rate for both groups.	7
Macle et al., 2015	Low risk of selection bias (randomization was done with permuted blocks of eight and the allocation sequence was computer-generated by an independent organization); low risk of performance bias (Patients were enrolled by study personnel and masked to their randomization assignment for the duration of the trial and study staff doing catheter ablations could not be masked to treatment allocation); low risk of detection bias (All efficacy and adverse outcomes were assessed by an independent adjudicating committee masked to treatment allocation); low risk of attrition bias (complete outcome for all the patients enrolled); low risk of reporting bias (we can find the research plan with “Adenosine following pulmonary vein isolation to target dormant conduction elimination (ADVICE): methods and rationale” though Pubmed); Unclear risk of other bias (insufficient information about other sources of bias).	–
Matsuo et al., 2010	Adequate case definition; consecutive series of cases; hospital controls; adequate information concerning the selection and definition of controls; groups controlled for all the baseline characteristics; ascertainment of outpatient exposure to adenosine text based on medical records for experiment groups and controls; patients not blinded to case–control status.; same non-Response rate for both groups.	7
Miyazaki et al., 2012	Adequate case definition; consecutive series of cases; hospital controls; adequate information concerning the selection and definition of controls; groups controlled for all the baseline characteristics; ascertainment of outpatient exposure to adenosine text based on medical records for experiment groups and controls; patients not blinded to case–control status.; same non-Response rate for both groups.	7
Cheung et al., 2013	Adequate case definition; consecutive series of cases; hospital controls; adequate information concerning the selection and definition of controls; groups controlled for the baseline characteristics are not mentioned; ascertainment of outpatient exposure to adenosine text based on medical records for experiment groups and controls; patients not blinded to case–control status.; same non-Response rate for both groups.	5
Lin et al., 2015	Adequate case definition; consecutive series of cases; hospital controls; adequate information concerning the selection and definition of controls; groups controlled for all the baseline characteristics; ascertainment of outpatient exposure to adenosine text based on medical records for experiment groups and controls; patients not blinded to case–control status.; same non-Response rate for both groups.	7

* DC, dormant conduction; ^*^Assessment of the quality of included studies according to Newcastle–Ottawa Scale for nonrandomized case–controls studies and the Cochrane Collaboration's tool for assessing risk of bias in randomized trials.

#### Long-term Success Rate of PVI Between Group A (+) and Group A (−)

**Figure 2:**
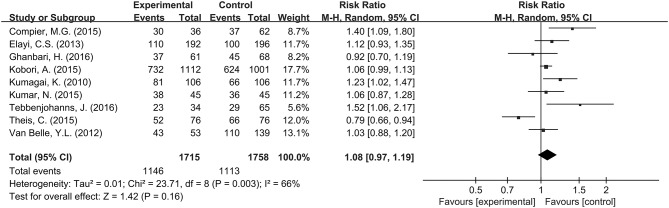
Forest plot comparing long-term success rates of PVI between Group A (+) and Group A (−).

**Figure 3:**
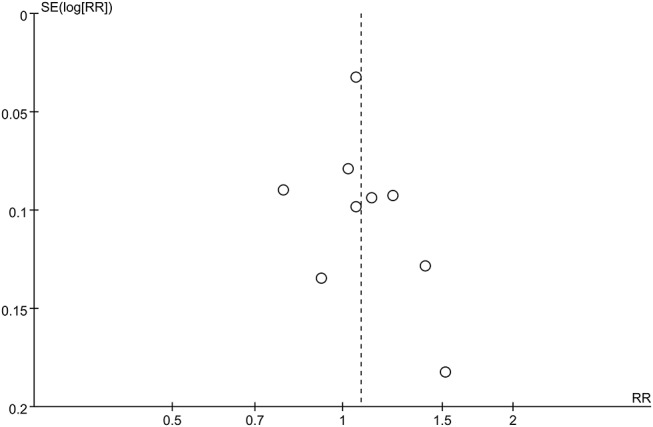
Funnel plot of standard errors against logarithms of odds ratios for studies comparing long-term success rates of PVI between Group A (+) and Group A (−).

The pooled meta-analysis demonstrated that there was no significant difference in freedom from recurrent AF between Group A (+) and Group A (−) (RR = 1.08, 95% CI: 0.97–1.19, *P* = 0.16, I^2^ = 66%; Figure 2). A funnel plot plotting standard errors against the logarithms of the RR are shown in Figure 3, demonstrating no significant publication bias.

#### Long-term Success Rate of PVI Between Group DC (+) and Group DC (−)

**Figure 4:**
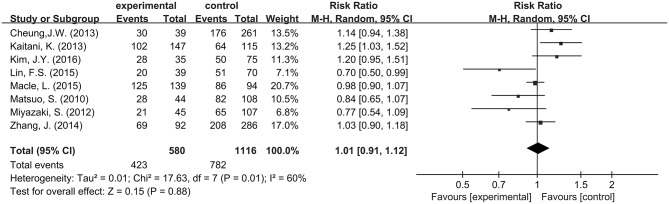
Forest plot comparing long-term PVI success rate between Group DC (+) and Group DC (−).

**Figure 5:**
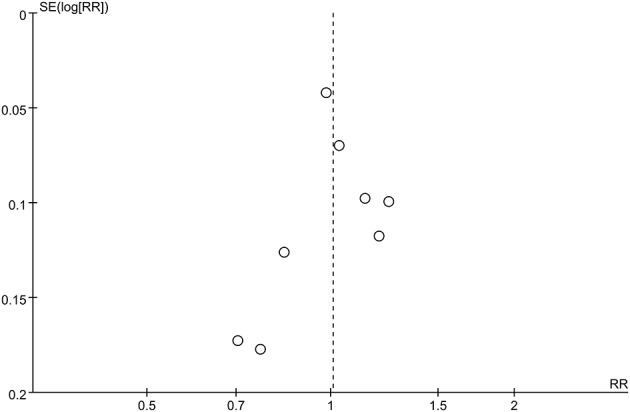
Funnel plot of standard errors against logarithms of odds ratios of studies comparing long-term PVI success rate between Group DC (+) and Group DC (−).

No significant difference was observed between Group DC (+) and Group DC (−) with a pooled RR of 1.01 (95% CI: 0. 91–1.12; *P* = 0. 88; I^2^ = 60%; Figure 4). A funnel plot plotting standard errors against the logarithms of the RR are shown in Figure 5, demonstrating no significant publication bias.

### Subgroup Analyses

**Figure 6:**
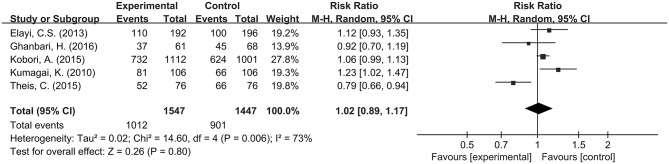
Subgroup analysis for CPVI.

**Figure 7:**
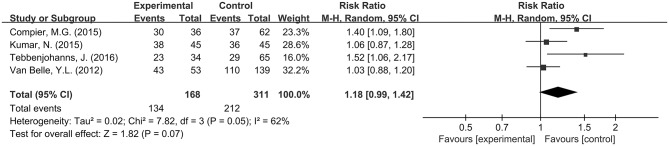
Subgroup analysis for CB-2G.

Additional subgroup analyses were performed for radiofrequency catheter ablation (RFCA) and CB-2G catheter ablation for PVI in Group A (+) and Group A (−). For RFCA, no difference in success rate was observed in Group A (+) and Group A (−) for patients with a RR of 1.02 (95% CI: 0.89–1.17; *P* = 0.80; Figure 6), which was accompanied by significant heterogeneity (I^2^ = 73%). Similarly, for CB-2G, success rates for those who underwent adenosine testing (*n* = 134) were not significantly different from those who did not have such a test (*n* = 212), with a pooled RR of 1.18 (95% CI = 0. 99–1.42; *P* = 0.07; Figure 7) with significant heterogeneity (I^2^ = 62%).

### Sensitivity Analysis

Sensitivity analysis included study design and adenosine test, and none of them showed significant interference with study outcomes. Results are shown in Table 4.

**Table 4:** Results of sensitivity analysis.

	**Studies**	**RR**	**95% CI**	*p*-value^*^	**Study heterogeneity**			
					**Chi^**2**^**	**df**	***I*^**2**^, %**	*p*-value^*^
**GROUP A (+) AND GROUP A (−)**								
RCT	3	0.92	0.75–1.14	0.46	10.10	2	80	0.006^*^
AO^*^	6	1.07	0.93–1.23	0.33	19.43	5	74	0.002^*^
PSAF^*^	4	1.01	0.82–1.24	0.95	15.05	3	80	0.002^*^
**GROUP DC (+) AND GROUP DC (−)**								
AO^*^	4	1.02	0.87–1.19	0.83	8.53	3	65	0.04^*^
PSAF^*^	3	0.98	0.90–1.08	0.70	2.61	2	23	0.27

* Significance values; ^*^AO, adenosine only; ^*^PSAF, paroxysmal atrial fibrillation.

## Discussion

Adenosine testing after AF ablation procedures has been widely adopted for demonstrating DCs, which are further ablated to reduce AF recurrence rates (Hocini et al., 2005). However, in our study, the result of pooled meta-analysis suggested that adenosine test may not help to reduce the long-term AF recurrence after CPVI, and further subgroup analysis also confirmed the result. Some recent studies also suggested negative result of adenosine test based on CPVI (Theis et al., 2015; Ghanbari et al., 2016). The reason might be explained by the mechanism of PVI re-connection after CPVI ablation dose not totally attributed by DCs (Linz et al., 2018). Potential mechanisms of AF recurrence after CPVI may due to failure of trans-mural injury of PVAs (Rostock et al., 2006), heterogeneity of myocardial sleeves extending into the pulmonary veins (Ho et al., 2001) or so on. As a consequence, the necessity and applicability of adenosine test diminished in the context of CPVI adoption ablation strategy and Whether other techniques, such as pacing along the PVI line by the distal tip of the ablation catheter to identify viable myocardium or potential gaps (Schaeffer et al., 2015) improves PVI outcome should be investigated in the future.

However, a recent meta-analysis has shown that long-term success rates of PVI were improved by further eliminating DCs that have been identified by adenosine test for patients underwent segmental ablation for AF (McLellan et al., 2017). The discrepancy results with the results of the previous meta-analysis (McLellan et al., 2017) may due to improved ablation strategies (Ouyang et al., 2004). The 3-dimensional Electroanatomical Mapping System for RFA provides better visualization and reduce the need for excessive ablation (Ouyang et al., 2004). Ablation strategies based on CPVI ablation, instead of segmental ablation, were comprehensively adopted for AF patients either paroxysmal AF or persistent AF, leading to better AF control in the long-term (Gepstein et al., 1997). Previous studies had shown that segmental ablation was inferior to long term treatment compared with CPVI, and leads to more complications, such as pulmonary stenosis (Oral et al., 2003). Additionally, cryo-application offers spherical contact with the PV autrum (PVA), guided by annular Achieve catheter and vasography, provided CPVI by the single-shot technique (Nakagawa et al., 2007). Consequently, modifying skills and appliances, meaningful of adenosine administration may have diminished the need for AF re-ablation.

Complications arising from ablating DCs could further contribute to the lack of efficacy. For example, excessive ablation creates scarring of the atrial myocardium, which can serve as substrates for re-entry (Pappone et al., 2004; Tse et al., 2016). Indeed, a previous study compared anatomically guided CPVI with wide atrial ablation, demonstrating that the latter approach significantly increased the likelihood of micro-reentry ablation by producing areas of conduction slowing and block (Hocini et al., 2005). Moreover, we found that fluoroscopic time and procedure time were prolonged due to adenosine administration.

## Limitations

This systematic review and meta-analysis has several potential limitations. There was moderate heterogeneity across the included studies, which may be due to the following factors. Firstly, differences in study participants between each study especially the types of AF, and in detection criteria were observed. Secondly, several studies have used additional methods during adenosine testing for provoking DCs, such as isoproterenol administration during adenosine test. Thirdly, the dose of adenosine, administration method and procedure (such as waiting period after adenosine) used to unmask dormant conduction was not uniform, this may affect the clinical outcomes. Fourthly, the successful rate of PVI may vary across medical centers due to variation in technical competencies, skills, and outcome measures. As such, the readers are advised to interpret the findings carefully. Nevertheless, funnel plot analysis revealed no significant publication bias. RCTs on CB-2G did not include a high number of participants and additional clinical trials are needed to confirm these findings.

## Conclusions

In conclusion, regular adoption of adenosine test could not further improve PVI success rate basing on long-term observation and elimination of DCs provoked by adenosine after CPVI did not significantly reduce AF recurrence after catheter ablation.

## Author Contributions

YX conceived and designed the study. YX and GT guided the study. CC and DL searched and screened studies independently and disagreements were resolved by adjudication with input from YX. XL, ZW, YL, and FZ helped finished the figures and tables. CC, DL, and GT finished the manuscript writing. JH and TL helped to refine the manuscript.

### Conflict of Interest Statement

The authors declare that the research was conducted in the absence of any commercial or financial relationships that could be construed as a potential conflict of interest. The handling editor is currently editing co-organizing a Research Topic with one of the authors GT, and confirms the absence of any other collaboration.

## Abbreviations

AF: atrial fibrillation
CPVI: circumferential pulmonary vein isolation
PVI: pulmonary vein isolation
DC: dormant conduction
PVs: pulmonary veins
